# G protein-coupled receptor 1 participating in the mechanism of mediating gestational diabetes mellitus by phosphorylating the AKT pathway

**DOI:** 10.1515/biol-2022-0920

**Published:** 2024-08-23

**Authors:** Yanbin Zhu, Shufeng Huang, Dan Chai, Lei Liang

**Affiliations:** Department of Obstetrics and Gynecology, Shenzhen Futian District Maternity & Child Healthcare Hospital, Shenzhen, 518017, Guangdong, China; Department of Gynecology, Shenzhen Qianhai Shekou Free Trade Zone Hospital, Shenzhen, 518067, Guangdong, China; Department of Obstetrics and Gynecology, Shenzhen Hospital of Southern Medical University, Shenzhen, 518000, Guangdong, China

**Keywords:** GPR1, AKT, GDM, diabetes treatment, pregnancy, metabolic dieases

## Abstract

Gestational diabetes mellitus (GDM) is a metabolic disease that occurs during pregnancy. Herein, we investigate G protein-coupled receptor 1 (GPR1) in mediating GDM through the phosphorylation of serine/threonine kinase (AKT) pathway. Thirty pregnant SD rats were grouped into: normal pregnancy control group (NC), GDM model group, and GDM model + high-dose GPR1 antagonist treatment (GDM + Ari) group. GDM model was established, and the GDM + Ari group adopted GPR1 antagonist aripiprazole. The blood glucose level, insulin level, and insulin resistance (IR) were detected. The expression and phosphorylation of GPR1, AKT, and extracellular signal-regulated kinase (ERK) in placental tissue were detected using reverse transcription-polymerase chain reaction (RT-PCR) and western blotting (WB). The serum insulin concentration, glucose concentration, and glycated hemoglobin concentration during pregnancy in GDM group SD rats were significantly higher than those in the NC group (*P* < 0.05). The expression and phosphorylation levels of GPR1, AKT, and ERK in the placental tissue of SD pregnant rats in the GDM group were significantly lower than those in the NC group. Furthermore, compared with the GDM group, the expression of GPR1, AKT, and ERK in placental tissue was significantly reduced in the GDM + Ari group, while simultaneously enhancing the blood glucose level and IR level. In addition, the survival number, body weight, and malformation rate of the offspring of the GDM + Ari group were significantly improved, and there was no significant effect on the number of offspring. The expressions of GPR1, AKT, and ERK in placental tissue exhibited a significant decrease, while the glucose level and IR were observed to increase in the GDM + Ari group. Enhancing the expression of GPR1 may activate AKT phosphorylation to alleviate GDM. GPR1 could potentially serve as a novel target for diabetes treatment, offering new insights into managing GDM.

## Graphical abstract

**Figure:**
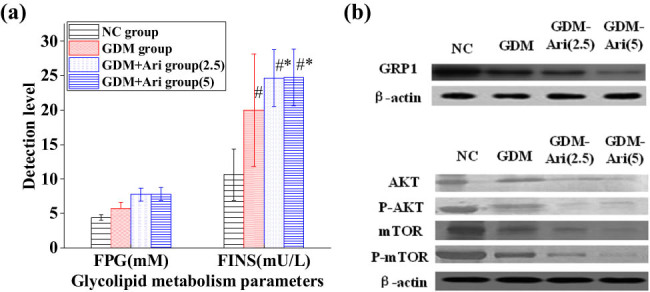

## 1 Introduction

Gestational diabetes mellitus (GDM) is a metabolic disease occurring during pregnancy and is characterized by glucose metabolism disorders that first appear during pregnancy, which can pose a serious threat to the health of pregnant women and infants [1]. In the 2021 global and regional GDM prevalence assessment report, the prevalence of GDM was as high as 27.0% in the Southeast Asia (SEA) region and 7.5% in the Middle East and North Africa (MENA) region [2]. The annual incidence of GDM in China accounts for 9.3% of the total increase in the world [3]. Current studies have shown that in addition to insulin resistance (IR) during pregnancy, there are many other factors affecting the occurrence of GDM, including genetic factors, lifestyle, abnormal insulin secretion, and fetal factors [4]. At present, many countries and regions around the world have developed special strategies and treatment programs for women, including nutritional intervention, insulin injection, and oral hypoglycemic drugs [5]. However, there are still many challenges in the accurate diagnosis and effective treatment of GDM.

With the continuous advancement of medical technology and the increasing emphasis on health awareness, the prevention and treatment of GDM are becoming increasingly refined. Currently, the management of GDM primarily involves a range of lifestyle interventions and pharmacotherapy [6]. In terms of lifestyle interventions, dietary and exercise programs have emerged as the cornerstone in managing GDM [7]. For pregnant women with severe diabetes, drug therapy has become an indispensable measure. The pathogenesis of GDM has a variety of mechanisms, including β-cell dysfunction, IR, adipose tissue dysfunction, gluconeogenesis, intestinal microbiota imbalance, and oxidative stress [8]. Many studies have confirmed that molecular markers, such as metabolites, single nucleotide polymorphisms (SNPs), microRNA (miRNA), and proteins in the mechanism of GDM, can supplement existing clinical risk factors to identify women at high risk of developing GDM during pregnancy and postpartum type 2 diabetes (T2D) [9]. Therefore, the search for new therapeutic targets has become a research hotspot in the prevention and treatment of GDM.

Phosphatidylinositol 3-kinase (PI3K)/protein kinase B (AKT) participates in various cellular processes, including glucose metabolism, insulin signaling, cell survival, and proliferation. AKT is a serine/threonine kinase that is activated by phosphorylation of upstream kinases such as PI3K and 3-phosphatidylinositol-dependent protein kinase 1 (PDK1) at threonine 308 and serine 473. Studies have shown that the AKT phosphorylation pathway has a major role in regulating insulin metabolism and cell proliferation [10]. In a study to explore the mechanism of microRNA-351/flotillin-2 (FLOT2)/PI3K/AKT and IR during pregnancy, GDM rats had increased levels of PI3K, AKT, and FLOT2, as well as phosphorylation of PI3K and AKT, while miR-351 expression was decreased [11]. Activated Akt enters and phosphorylates many of its downstream substrates to regulate the severity of IR, thereby affecting the occurrence of GDM, suggesting that the symptoms of GDM rats can be regulated through the PI3K/AKT pathway. G protein-coupled receptor (GPR1) can regulate cell metabolism, proliferation, and survival by activating the PI3K-AKT signaling pathway. Studies have found that GPR1 expression promotes the phosphorylation of Akt and ERK and the expression of proliferating cell nuclear antigen (PCNA) protein in choriocarcinoma trophoblast cells [12]. Mlyczyńska et al. [13] found that GPR1 was expressed in the pituitary gland of pigs during the estrous cycle and early pregnancy and proposed that GPR1 was related to the regulation of MAPK/Erk1/2, Akt, and AMPK signaling pathways affecting reproductive function. Therefore, this article explored GPR1 in the pathogenesis of GDM and its interaction with the AKT pathway through animal experiments.

## 2 Materials and methods

### 2.1 Subjects

Sprague–Dawley (SD) rats (Changzhou Cavens Experimental Animal Co., Ltd.), SPF grade, all of them 8–9 weeks old, weighing (186 ± 9) g, were used in the experiments. The rats were kept in a general animal room at a temperature of 24–27°C, a relative humidity of 60%, and the alternating light cycle was parallel to the outside day and night. Light and dark exchange was carried out every 12 h. The purchased rats were adaptively housed for 1 week, and all rats were allowed to eat and drink freely.


**Ethical approval**: The research related to animal use complied with all the relevant national regulations and institutional policies for the care and use of animals and has been approved by the Institutional Animal Care and Use Committee (IACUC).

### 2.2 GDM model and grouping

The procured SD rats were placed in a cage overnight at a ratio of 2:1 female to male. On the second day, the vaginal secretions of the female rats were taken and observed under a microscope, and the presence of sperm was judged as pregnancy, marked as day 0 of pregnancy (d0). Eighteen pregnant SD rats were randomly grouped: NC, GDM model, and GDM model + high-dose GPR1 antagonist treatment (GDM + Ari).

For GDM model establishment, GDM rats were intraperitoneally injected with 45 mg/kg streptozotocin (STZ), and blood glucose was collected from the tail vein 24 h after administration. When the blood glucose concentration was greater than 11.1 mM, the model was considered successful.

NC group had no interventions.

In the GDM + Ari group, GDM rats were intraperitoneally injected with GPR1 antagonist aripiprazole at 2.5 mg/(kg day)^−1^, 5 mg/(kg day)^−1^ for 3 weeks, and the rats were killed after continuous injection. The blood collection and dissection were performed.

### 2.3 Serum glycosylated hemoglobin (HbA1c)

Blood samples were collected in a fasting state in the morning before modeling and 24 h after modeling. The content of HbA1c was detected by high-performance liquid chromatography.

Methods: The collected blood was stored in ethylenediamine tetraacetic acid (EDTA) anticoagulant tube (−80°C). Before the test, the blood was taken out of the refrigerator and placed in an ice box for dissolution. Then, part of the blood was put into a centrifuge tube for centrifugation (3,000 r/15 min), the serum was separated, and normal saline (volume 1:5) was added to the serum for washing. After 4 h, the blood was centrifuged (1,000 r/10 min). After removal of the supernatant, 2 nM EDTA-Na_2_ was added to the centrifuge tube, shaken for 5 min, and then centrifuged (3,000 r/5 min). The upper blood red liquid, HbA1c, was sucked by using a pipette. American Bio-Rad D-10 HbA1c instrument was applied.

### 2.4 Glucose and lipid metabolism parameters of pregnant SD rats after modeling

Following modeling, pregnant SD rats were subjected to tail vein blood sampling in the morning under a fasting state, and the rats were fasted the night before blood sampling.

Fasting plasma glucose (FPG) was detected by using the Johnson & Johnson One Touch Horizon blood glucose meter and matching test paper. The tail (5 mm) of pregnant SD rats was removed, and the hand was used to massage until bleeding. The test paper dipped into the blood was put into the blood glucose meter to detect FPG.

Fasting insulin (FINS) was detected by enzyme-linked immunosorbent assay (ELISA): a rat FINS ELISA kit was used for detection, and the operation was carried out in strict accordance with the instructions.

- The required reagents were mixed based on the instructions, eliminating bubbles in the liquid.
- About 50 μL of the standard was added to the blank microplate, repeating five wells.
- Samples to be tested were numbered, and then 50 μL of samples were added to the wells of the blank microplate, repeating five wells.
- Immediately, 50 μL of the enzyme labeling solution was added to the reaction empty well, shaken well with an oscillator, and incubated in the cell incubator for 1 h.
- The liquid in the microplate was shaken until clear and washed with washing solution. It was shaken for 30 s, and the washing solution was dry; this was repeated three times.
- Substrate was added to each sampling well, with 50 μL of solution A and 50 μL of solution B, and incubated in the dark (room temperature, 10 min) after uniform shaking.
- The microplate was removed, 50 μL termination solution was added, and the microplate was immediately placed in the instrument to detect the absorbance of the sample (450 nm).
- The standard curve was drawn. The vertical coordinate is the absorbance value, and the abscissa is the concentration of the standard. The concentration of the sample to be tested was calculated.

For assessment of IR-related indicators, IR index, and islet B cell function, homeostasis model assessment (HOMA) was adopted.

### 2.5 RT-PCR to detect gene mRNA expression levels

The placentas of pregnant SD rats were collected. The mRNA expression levels of GPR1, AKT, P-AKT, mammalian target of rapamycin (mTOR), and P-mTOR were detected by RT-PCR.

Total RNA was extracted from the placental tissue using the Trizol method. Labeled Eppendorf (EP) tubes were placed in an ice box, mashed tissue was placed into the EP tubes, thoroughly ground, centrifuged (3,000 r/10 min), and the supernatant was collected. About 250 μL of chloroform was added to the EP tube containing the supernatant, shaken to make it fully react, and centrifuged after 3 min to collect the supernatant. Isopropyl alcohol (volume 1:5) was added to extract RNA, shaken thoroughly, incubated at −20°C for the reaction, and centrifuged to collect the precipitate after 15 min. The precipitate was collected by centrifugation (1,200 r/5 min), and RT-PCR grade water (AM9935) was added to dissolve the precipitate. The concentration and purity of the RNA were detected by the instrument. The obtained RNA was reverse-transcribed into cDNA, and the PCR quantitative kit was used for the experiment. The reference gene GAPDH, F: 5′-CTGGAGAAACCTGCCAAGTATG-3′, R: 5′-GGTGGAAGAATGGGAGTTGCT-3′; primers for GPR1, AKT, P-AKT, and mTOR were designed.

GPR1 primer: F: 5′ CCACAGGTTTCTTCATTCTGCAC - 3′, R: 5′ GCCTCACACAAAAGCTGTATACTGAC - 3′.

AKT primer: F: 5′-GGACAACCGCCATCCAGACT-3′, R: 5′-GCCAGGGACACCTCCATCTC-3′.

P-AKT primers: F: 5′-GTGCTGGAGGACAATGACTACGG-3′, R: 5′-AGCAGCCCTGAAAGCAAGGA-3′.

mTOR primers: F: 5′-ATGACGAGACCCAGGCTAAG-3′, R: 5′-GCCAGTCCTCTACAATACGC-3′.

### 2.6 Western blotting (WB) to detect the protein expression level

Part of the placenta tissues of the rats were cut and placed in the corresponding EP tube. It was ground thoroughly, adding 200 μL of protein lysate, and then it was mashed to homogenize. The placenta tissues were centrifuged (4°C, 3,000 r/15 min), and the supernatant was collected. The protein concentration was detected using the bicinchoninic Acid Assay (BCA), and the operation was strictly according to the BCA protein concentration assay kit. The concentration and separation gels were prepared using sodium dodecyl-sulfate polyacrylamide gel electrophoresis (SDS-PAGE). The collected proteins were subjected to gel electrophoresis, then transferred to polydivinylidene fluoride (PVDF) membrane, washed using the buffer solution (TBST) and blocked by adding 5% skim milk powder. The primary antibody was added 30 min later, incubated at 4°C, and it was placed in a shaker overnight. The membrane was washed again with TBST, repeating three times. The secondary antibody was added and incubated at room temperature, the membrane was rinsed, and then the infrared imager was used for development. The gray value was analyzed.

### 2.7 Hematoxylin and eosin (HE) staining and immunohistochemistry

First, the placental tissues of rats were dehydrated, with the alcohol gradient concentration from low to high. The placentas were soaked in xylene to make the tissues transparent. The clear tissue was immersed in paraffin for embedding, and the wax solution was solidified before use. The paraffin specimens were sectioned using a micrograph and placed in a water bath at 40°C to stretch the wax slides. Then, they were removed and placed in the center of the slide and baked at 60°C for 3 h. The dried sections were deparaffinized and stained with HE. First, the slices were stained with hematoxylin and washed with water after 5 min. The paraffin sections turned blue. Then, they were stained with eosin, soaked in water for 30 s, 5 min later, and then paraffin sections were dehydrated with alcohol. They were transparent again with xylene, sealed, and examined under a microscope. The photographic results were analyzed according to the degree of staining and the positive range.

### 2.8 Relevant equations

The relevant equations are as follows:

(1) \[{{\mathrm{purity}}}_{{\mathrm{RNA}}}=A260/A280=2.0,]\]

(2) \[{C}_{{\mathrm{RNA}}}={\mathrm{OD}}260\times 40\times 10{\mathrm{-}}3\left({\mathrm{\mu }}{\mathrm{g}}/{\mathrm{\mu }}{\mathrm{L}}),]\]

(3) \[{\mathrm{HOMA}}\text{-}{\mathrm{IR}}={\mathrm{FINS}}\times {\mathrm{FBG}}/22.5,\hspace{1em}]\]

(4) \[{\mathrm{HOMA}}\text{-}{\mathrm{\beta }}=20\times {\mathrm{FINS}}\left/\left({\mathrm{FBG\; -}}3.5).]\]

### 2.9 State of offspring

The 21-day survival rate and fetal malformation rate of the offspring in the NC group, GDM group, GDM + Ari(2.5) group, and GDM + Ari(5) group were observed, and the number of fetuses and birth weight were recorded.

### 2.10 Data processing

SPSS 25.0 data analysis software was utilized for statistical analysis. Measurement data are presented as the mean ± standard deviation, and a *t-*test was employed for analysis. Count data are expressed as percentages (%), and the *X* ^2^ test was used for analysis. Statistical significance was defined as a *P* value less than 0.05.

## 3 Results

### 3.1 Contrast of blood glucose levels before and after modeling in pregnant SD rats

**Figure 1:**
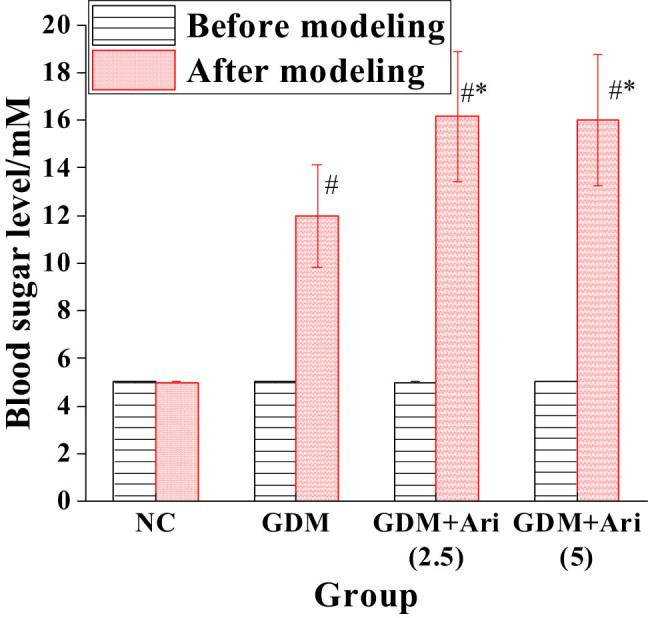
Contrast of blood glucose levels before and after modeling. Note: relative to controls, ^#^ *P <* 0.05; compared with the GDM group, *P < 0.05. NC: control group; GDM: gestational diabetes mellitus model; GDM + Ari(2.5): indicates treatment with 2.5 mg/(kg day)^−1^ GPR1 antagonist in the GDM model; GDM + Ari(5): indicates treatment with 5 mg/(kg day)^−1^ GPR1 antagonist in the GDM model. The blood glucose levels in the GDM group were significantly elevated, indicating successful model establishment, whereas in the GDM + Ari group, the blood glucose concentration was even higher, suggesting that the GPR1 antagonist could exacerbate IR in GDM rats.

The blood glucose level of the GDM group was markedly higher than that of NC after modeling. The blood glucose level of the GDM + Ari group was markedly higher than that of NC after GPR1 antagonist administration. Moreover, the blood glucose levels in the GDM + Ari group were significantly higher compared to the GDM group (*P* < 0.05), with statistically significant differences (Figure 1).

### 3.2 Contrast of HbA1c levels in pregnant SD rats after modeling

**Figure 2:**
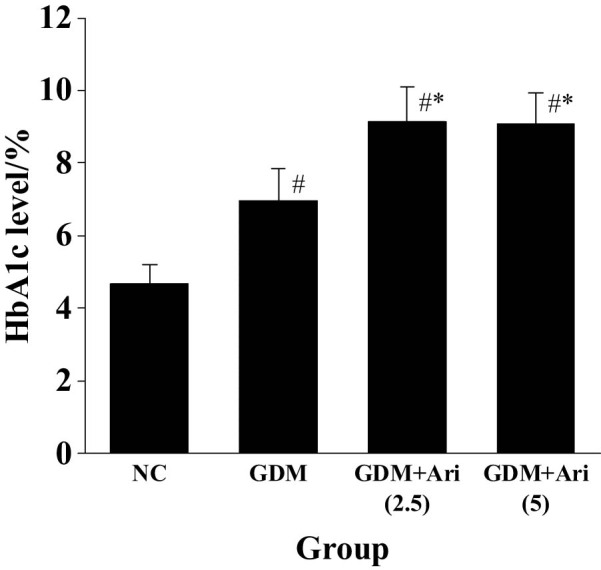
Contrast of HbA1c levels after modeling. Note: Relative to controls, ^#^ *P <* 0.05; compared with the GDM group, *P < 0.05. NC: control group; GDM: gestational diabetes mellitus model; GDM + Ari(2.5): indicates treatment with 2.5 mg/(kg day)^−1^ GPR1 antagonist in the GDM model; GDM + Ari(5): indicates treatment with 5 mg/(kg day)^−1^ GPR1 antagonist in the GDM model; HbA1c represents glycated hemoglobin, which reflects blood glucose levels.

The HbA1c level of the GDM group was markedly superior than in the NC after modeling, and that of the GDM + Ari group was superior than in the NC after GPR1 antagonist administration (*P <* 0.05). Moreover, the HbA1c levels in the GDM + Ari group were significantly higher compared to the GDM group (*P* < 0.05), with statistically significant differences (Figure 2).

### 3.3 Contrast of glucose and lipid metabolism parameters in pregnant SD rats

**Figure 3:**
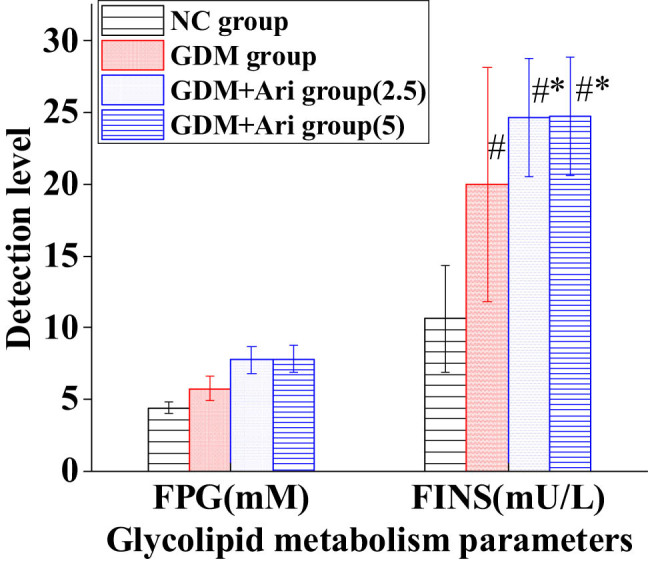
Contrast of glucose and lipid metabolic parameters. Note: Relative to controls, ^#^ *P <* 0.05; compared with the GDM group, *P < 0.05. NC: control group; GDM: gestational diabetes mellitus model; GDM + Ari(2.5): indicates treatment with 2.5 mg/(kg day)^−1^ GPR1 antagonist in the GDM model; GDM + Ari(5): indicates treatment with 5 mg/(kg day)^−1^ GPR1 antagonist in the GDM model; FPG represents fasting plasma glucose; FINS represents fasting insulin.

Figure 3 illustrates that the FPG level of GDM and GDM + Ari groups was markedly higher than in the NC (*P <* 0.05). The level of FINS in the GDM group was markedly higher than in NC. Relative to the NC group, the level of FINS in the GDM + Ari group was markedly increased (*P <* 0.05). Furthermore, the FPG and FINS levels in the GDM + Ari group were significantly higher compared to the GDM group (*P* < 0.05), with statistically significant differences.

### 3.4 Contrast of HOMA in pregnant SD rats

**Figure 4:**
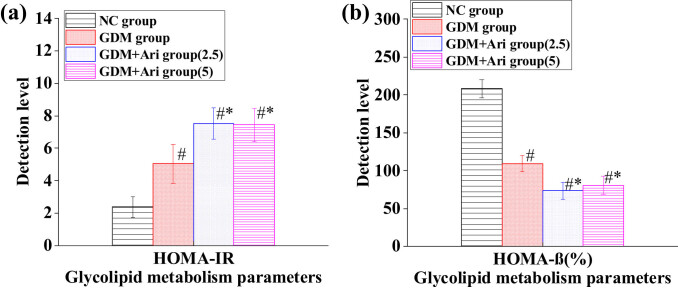
Contrast of HOMA: (a) HOMA-IR index and (b) HOMA-β index. Note: Relative to controls, ^#^ *P <* 0.05; compared with the GDM group, *P < 0.05. NC: control group; GDM: gestational diabetes mellitus model; GDM + Ari(2.5): indicates treatment with 2.5 mg/(kg day)^−1^ GPR1 antagonist in the GDM model; GDM + Ari(5): indicates treatment with 5 mg/(kg day)^−1^ GPR1 antagonist in the GDM model; HOMA-IR represents the IR index, where HOMA-IR > 2.6 indicates IR; HOMA-β is used to evaluate pancreatic β-cell function.

Figure 4 indicates that the HOMA-IR index of pregnant rats in the GDM and GDM + Ari groups was markedly higher than in the NC (*P <* 0.05). Furthermore, the HOMA-IR in the GDM + Ari group was significantly higher compared to the GDM group (*P* < 0.05), with statistically significant differences. The HOMA-β index in the GDM and GDM + Ari groups was markedly lower as than in the NC (*P <* 0.05). Additionally, the HOMA-β index in the GDM + Ari group was significantly lower compared to the GDM group (*P* < 0.05), with statistically significant differences.

### 3.5 HE staining characteristics of pregnant SD rats

**Figure 5:**
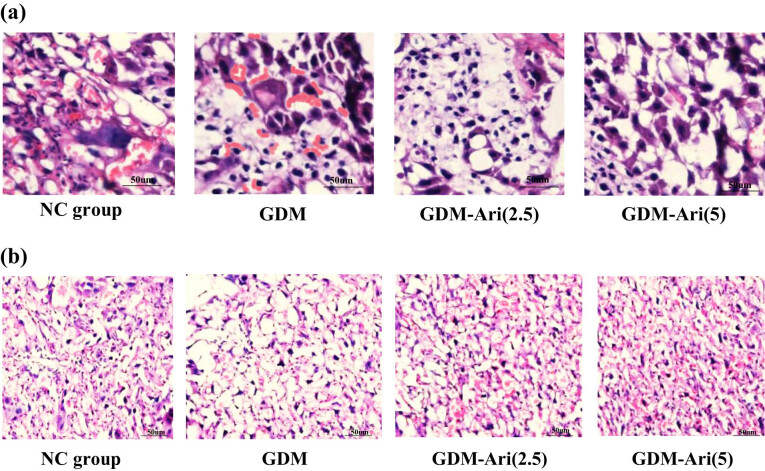
Histological images of placental tissues from SD pregnant rats stained with hematoxylin and eosin (HE) (a) HE staining, magnification ×200, and immunohistochemistry. (b) Immunohistochemical staining, magnification ×200. Note: NC: control group; GDM: gestational diabetes mellitus model; GDM + Ari(2.5): indicates treatment with 2.5 mg/(kg day)^−1^ GPR1 antagonist in the GDM model; GDM + Ari(5): indicates treatment with 5 mg/(kg day)^−1^ GPR1 antagonist in the GDM model.

The results showed that in the NC, the villi were smaller in volume, the number of trophoblast cells was less, the syncytiotrophoblast cells were densely aggregated into trophoblast cell knots, and the trophoblast cells did not show delamination. The villous interstitium was tightly integrated, the number of venule vessels was more, and fibrin was deposited in the intervillous space. In the GDM group, the villus maturation was different, and most of the villi were poorly developed. The number of trophoblast cells and syncytiotrophoblast knots increased markedly, and the basement membrane of trophoblast cells thickened locally. The fibrotic portion of the villous interstitial substance increased, the villous state became edematous, and the capillaries distended. In the GDM + Ari group, there was a significant increase in the thickness of the trophoblast basement membrane in pregnant mouse placentas, accompanied by a noticeable reduction in lipid droplet accumulation within the placenta (Figure 5).

### 3.6 Expression of GPR1 mRNA in placental tissue of pregnant SD rats

**Figure 6:**
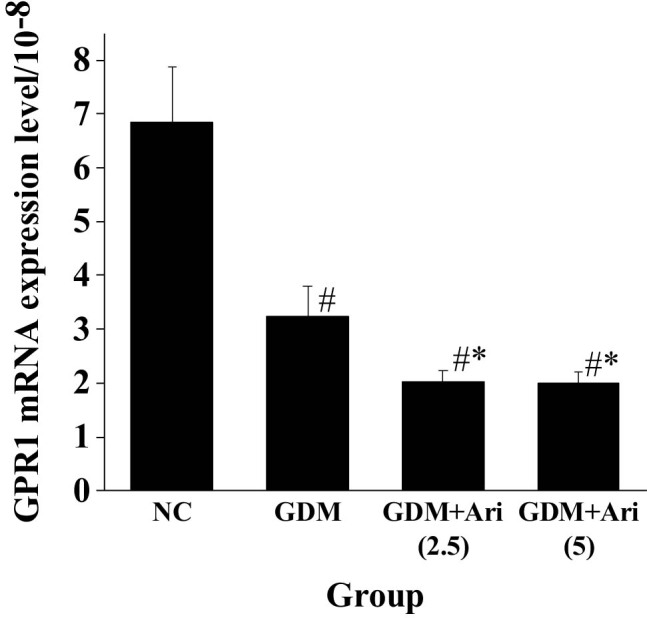
GPR1 mRNA expression level in the placental tissue. Note: As against controls, ^#^ *P <* 0.05; compared with the GDM group, *P < 0.05. NC: control group; GDM: gestational diabetes mellitus model; GDM + Ari(2.5): indicates treatment with 2.5 mg/(kg day)^−1^ GPR1 antagonist in the GDM model; GDM + Ari(5): indicates treatment with 5 mg/(kg day)^−1^ GPR1 antagonist in the GDM model; GPR1 mRNA represents the transcription level of GPR1.

Figure 6 indicates that the GPR1 mRNA expression level of pregnant rats in the GDM and GDM + Ari groups was markedly lower than in the NC (*P <* 0.05). Furthermore, the GPR1 mRNA expression in the GDM + Ari group was significantly lower compared to the GDM group (*P* < 0.05), with statistically significant differences.

### 3.7 Expression of AKT, P-AKT, mTOR, and P-mTOR mRNA in placental tissues of pregnant SD rats

**Figure 7:**
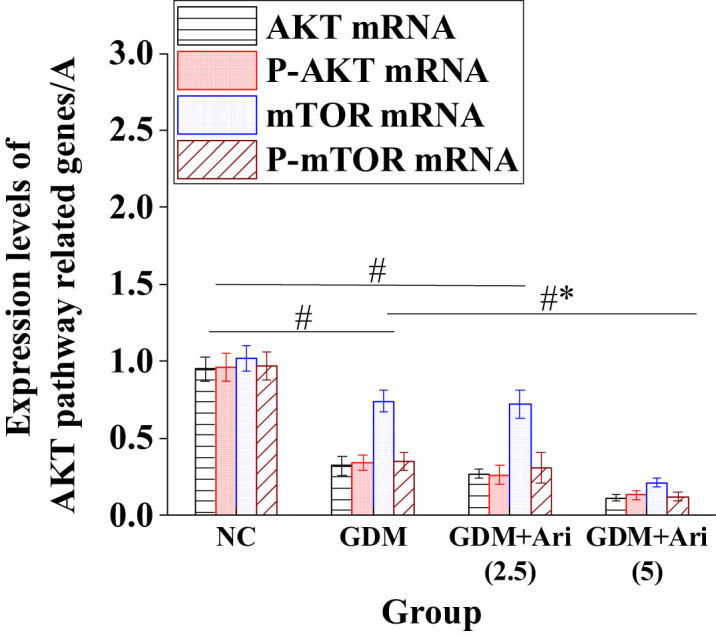
Expression of AKT pathway-related proteins in the placental tissues. Note: As against controls, ^#^ *P <* 0.05; compared with the GDM group, *P < 0.05. NC: control group; GDM: gestational diabetes mellitus model; GDM + Ari(2.5): indicates treatment with 2.5 mg/(kg day)^−1^ GPR1 antagonist in the GDM model; GDM + Ari(5): indicates treatment with 5 mg/(kg day)^−1^ GPR1 antagonist in the GDM model; AKT: protein kinase B; P-AKT: phosphorylated protein kinase B; mTOR: mammalian target of rapamycin; P-mTOR: phosphorylated mammalian target of rapamycin.

Figure 7 indicates that the expression levels of P-AKT mRNA and P-mTOR mRNA in GDM and GDM + Ari groups were apparently lower relative to NC (*P <* 0.05). Moreover, the levels of AKT mRNA and mTOR mRNA were significantly decreased compared to the NC group (*P* < 0.05), with statistically significant differences. Additionally, in the high-dose GDM + Ari group, the mRNA levels of AKT, P-AKT, mTOR, and P-mTOR in placental tissues were significantly reduced compared to the GDM group (*P* < 0.05).

### 3.8 GPR1 protein expression in placental tissue of pregnant SD rats

**Figure 8:**
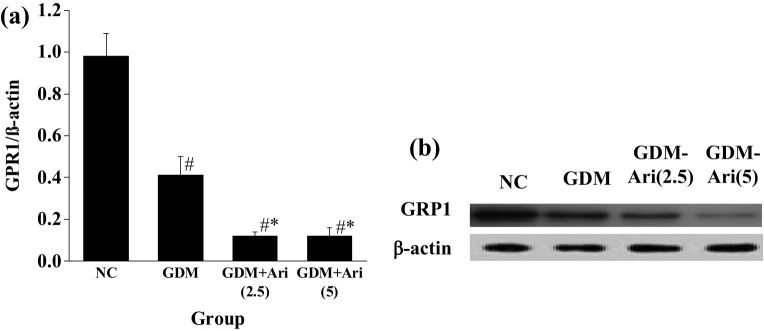
GPR1 protein expression in placental tissue. (a) protein expression level; and (b) protein expression gel image. Note: As against controls, ^#^ *P <* 0.05; compared with the GDM group, *P < 0.05. NC: control group; GDM: gestational diabetes mellitus model; GDM + Ari(2.5): indicates treatment with 2.5 mg/(kg day)^−1^ GPR1 antagonist in the GDM model; GDM + Ari(5): indicates treatment with 5 mg/(kg day)^−1^ GPR1 antagonist in the GDM model; GPR1 represents G protein-coupled receptor.

Figure 8 indicates that the expression level of GPR1 in the GDM and GDM + Ari groups was apparently lower than in the NC (*P <* 0.05). Furthermore, the expression level of GPR1 in pregnant rats of the GDM + Ari group was significantly lower compared to the GDM group (*P* < 0.05).

### 3.9 Expression of AKT, P-AKT, mTOR, and P-mTOR proteins in placental tissues of pregnant SD rats

**Figure 9:**
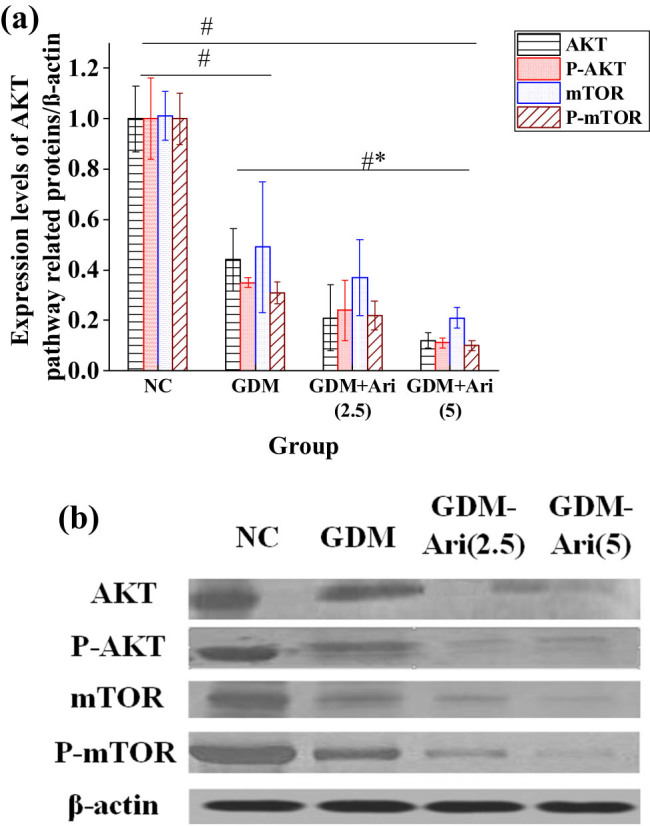
Contrast of AKT pathway-related protein expression levels in the placental tissue. (a) protein expression level; and (b) protein expression gel image. Note: As against controls, ^#^ *P <* 0.05; compared with the GDM group, *P < 0.05. NC: control group; GDM: gestational diabetes mellitus model; GDM + Ari(2.5): indicates treatment with 2.5 mg/(kg day)^−1^ GPR1 antagonist in the GDM model; GDM + Ari(5): indicates treatment with 5 mg/(kg day)^−1^ GPR1 antagonist in the GDM model; AKT: protein kinase B; P-AKT: phosphorylated protein kinase B; mTOR: mammalian target of rapamycin; P-mTOR: phosphorylated mammalian target of rapamycin.

Figure 9 reveals that the expression levels of P-AKT and P-mTOR in GDM and GDM + Ari groups were apparently lower than those in NC (*P <* 0.05). Furthermore, the expression levels of AKT, P-AKT, mTOR, and P-mTOR in pregnant rats of the GDM + Ari group were significantly lower compared to the GDM group (*P* < 0.05).

### 3.10 21-day survival rate and fetal malformation rate of offspring in each group

**Figure 10:**
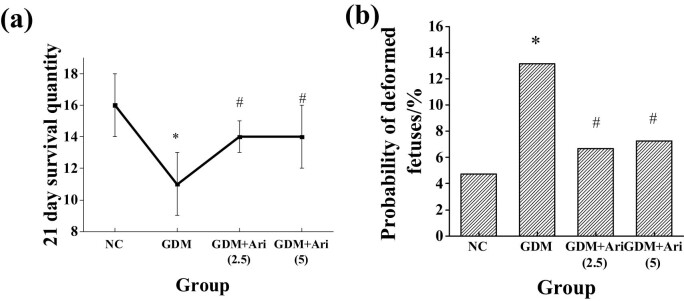
The 21-day survival number and fetal malformation rate of offspring in each group: (a) 21-day survival number and (b) malformation rate. *indicates a significant difference compared with NC (*P* < 0.05); #indicates a significant difference compared with GDM (*P* < 0.05).

The effects of the GPR1 antagonist on the 21-day survival number and malformation rate of the offspring on this pathway were observed, and the results are illustrated in Figure 10. Compared with the NC group, the survival rate of offspring significantly decreased, and the malformation rate significantly increased in the GDM group at 21 days. Compared with the GDM group, the 21-day survival number was obviously higher, and the malformation rate was significantly reduced in the GDM + Ari(2.5) and GDM + Ari(5) groups, with significant differences.

### 3.11 Number and weight of offspring at birth

**Figure 11:**
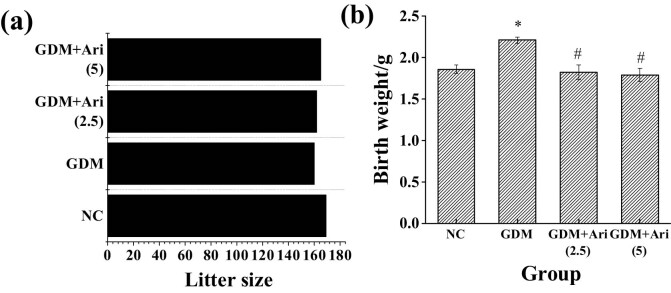
Contrast of number and weight of offspring at birth: (a) number of offspring and (b) weight of offspring at birth. *indicates a significant difference compared with NC (*P* < 0.05); #indicates a significant difference compared with GDM (*P* < 0.05).

The effects of the GPR1 antagonist on the number and weight of offspring were observed, and the results are illustrated in Figure 11. There was no significant difference in the number of offspring in each group (*P* > 0.05). The GDM group had significantly higher birth weight than the NC group, and the GDM + Ari(2.5) and GDM + Ari(5) groups had significantly lower birth weight (*P* < 0.05).

## 4 Discussion

GDM refers to a type of diabetes that occurs during pregnancy. The prevalence of GDM is on the rise worldwide [14], and it can have adverse effects on the pregnancy of patients and may lead to a series of complications. Patients with GDM exhibit elevated blood glucose levels during pregnancy, which, if left uncontrolled, can result in fetal macrosomia, fetal malformation, fetal death, and other complications [15]. The hyperglycemic environment exposes the fetus to excessive glucose levels and stimulates increased insulin secretion. This leads to excessive fat accumulation in the fetal body and subsequently contributes to the development of macrosomia while also impacting fetal organ function and increasing the risk of fetal malformations. Furthermore, GDM not only poses adverse effects on the fetus but also elevates the mother’s susceptibility to T2D [16]. Studies have shown that pregnant women with GDM have an apparently increased risk of developing T2D in the first 10 years of pregnancy [17]. This is because IR increases during GDM, and the insulin secreted by the islet β cells cannot meet the demand, causing the occurrence of hyperglycemia. If blood glucose is not controlled during pregnancy, it may accelerate the deterioration of islet β-cell function and increase the risk of diabetes. In addition, GDM can also increase the risk of complications such as gestational hypertension and preeclampsia, and the hyperglycemic environment impairs the vasoconstrictor function of pregnant women, leading to increased blood pressure [18]. Preeclampsia is a grave pregnancy complication that can result in life-threatening consequences for both the mother and the baby. Hence, GDM not only poses detrimental effects on maternal health but also escalates the risk of complications for both mothers and infants. Timely diagnosis and effective management of GDM are imperative to safeguard the well-being of pregnant women and fetuses.

This study used STZ to induce GDM. STZ can selectively destroy islet β cells, resulting in reduced insulin secretion, thus mimicking the pathological process of GDM [19]. The advantage of using STZ to induce GDM is that the experimental conditions can be controlled, making the experimental results more reliable. Herein, a series of experiments have been conducted adopting STZ-induced GDM animal models. In an experiment to explore whether crocetin can inhibit GDM in diabetic rats by inhibiting inflammatory response, an STZ-induced GDM model was adopted. By detecting indicators such as blood glucose level, insulin level, and islet morphology, researchers evaluated the occurrence and development process of GDM and the influence of different factors on GDM [20].

The occurrence of GDM is closely related to IR. Increased hormone levels during pregnancy lead to increased IR, which prevents insulin from working properly. IR refers to the decreased response of tissue cells to insulin, leading to the inability of insulin to effectively promote the uptake and utilization of glucose and then causing the occurrence of hyperglycemia [21]. In GDM patients, GPR1 participates in the mechanism of regulating IR by phosphorylating the AKT pathway. AKT is an important signaling molecule that participates in important physiological processes. In the process of insulin signaling, the activation of AKT can promote the translocation of intracellular glucose transporter (GLUT), increase glucose uptake and utilization, and thus reduce blood glucose levels. In a study, FPG, CRP, and PI3K/Akt were analyzed by qRT-PCR, morphological observation, and WB. This indicates that the inhibition of PI3K/Akt can accelerate the progression of GDM [22]. The research findings indicate that diabetic pregnancies are associated with reduced placental p-Akt levels and decreased mTOR phosphorylation. Moreover, under OGD conditions, primary cell cultures from the trophoblast layer demonstrate lower levels of p-Akt and p-mTOR compared to cells cultured under standard conditions (all *P* < 0.05). However, the effects of increased glucose concentration on their respective levels are opposite. Administration of AMPK inhibitors does not significantly affect Akt phosphorylation but partially reverses mTOR phosphorylation [23,24]. This suggests that Akt is involved in regulating mTOR activity in the trophoblast layer of the GDM placenta. In the study by Lees et al. [25], Pls-mediated ERK phosphorylation was found to be suppressed in GPR61. This is consistent with the results in this article, in which GPR1 expression levels were apparently decreased in pregnant GDM rats, and phosphorylated AKT levels were correspondingly decreased. This implies that the activity of the GPR1-AKT pathway is inhibited in pregnant GDM rats, leading to the blockage of insulin signaling. Further studies demonstrated that downregulation of GPR1 expression and inhibition of AKT phosphorylation effectively mitigate the development of GDM in pregnant rats [26]. Activation of the GPR1-AKT pathway enhances insulin signaling and facilitates glucose uptake and utilization, thereby ameliorating the symptoms associated with GDM. These findings suggest a close association between GDM occurrence and IR, with GPR1 playing a role in regulating GDM through AKT phosphorylation. Activation of GPR1 can enhance insulin signaling, thereby improving IR. In GDM pregnant rats, the decreased expression level of GPR1 significantly reduces AKT phosphorylation, consequently inhibiting insulin signaling transduction. Further studies indicate that by inhibiting GPR1 expression and AKT phosphorylation, the diabetic condition in GDM pregnant rats can be effectively ameliorated. Activation of the phosphorylated AKT pathway can enhance insulin signaling transduction and promote glucose uptake and utilization, thus alleviating diabetic symptoms. In subsequent studies, it was found that GPR1 antagonists did not affect the number of offspring produced in experimental pregnant rats and had positive significance in improving the survival, weight, and malformation of offspring. Based on the mechanism of GPR1 activation of the phosphorylated AKT pathway, therapeutic strategies targeting this pathway can be developed. This may contribute to drug development, gene therapy, or other interventions aimed at restoring insulin signaling transduction and alleviating diabetic symptoms.

## 5 Conclusion

GDM is a disease that can adversely affect both the mother and the fetus and may lead to a range of complications. An animal model of STZ-induced GDM can effectively simulate the pathological process of GDM. The occurrence of GDM is closely correlated with IR, and GPR1 participates in the mechanism of regulating IR by phosphorylating the AKT pathway. Therefore, further study on the regulatory mechanism of GPR1 on IR will help to understand the occurrence and development of GDM.

## References

[1] Mora-Janiszewska O, Faryniak-Zuzak A, Darmochwał-Kolarz D. Epigenetic links between microbiota and gestational diabetes. Int J Mol Sci. 2022;23(3):1831. 10.3390/ijms23031831 PMC8837149 35163753

[2] Wang H, Li N, Chivese T, Werfalli M, Sun H, Yuen L, et al. IDF diabetes atlas: estimation of global and regional gestational diabetes mellitus prevalence for 2021 by international association of diabetes in pregnancy study group’s criteria. Diabetes Res Clin Pract. 2022;183:109050 (1–7). 10.1016/j.diabres.2021.109050 34883186

[3] Ramezani Tehrani F, Behboudi-Gandevani S, Farzadfar F, Hosseinpanah F, Hadaegh F, Khalili D, et al. A cluster randomized noninferiority field trial of gestational diabetes mellitus screening. J Clin Endocrinol Metab. 2022;107(7):e2906–20. 10.1210/clinem/dgac181 35325164

[4] Amrom D, Schwartz SS. Maternal metabolic health, lifestyle, and environment - Understanding how epigenetics drives future offspring health. Curr Diabetes Rev. 2023;19(2):e220422203919. 10.2174/1573399818666220422085016 35466879

[5] Vasile FC, Preda A, Ștefan AG, Vladu MI, Forțofoiu MC, Clenciu D, et al. An update of medical nutrition therapy in gestational diabetes mellitus. J Diabetes Res. 2021;2021:5266919 (1–10). 10.1155/2021/5266919 PMC8616668 34840988

[6] Leslie RD, Ma RCW, Franks PW, Nadeau KJ, Pearson ER, Redondo MJ. Understanding diabetes heterogeneity: Key steps towards precision medicine in diabetes. Lancet Diabetes Endocrinol. 2023;11(11):848–60. 10.1016/S2213-8587(23)00159-6 37804855

[7] Ou L, Zhu Z, Hao Y, Li Q, Liu H, Chen Q, et al. 1,3,6-Trigalloylglucose: A novel potent anti-Helicobacter pylori adhesion agent derived from aqueous extracts of Terminalia chebula Retz. Molecules (Basel, Switz). 2024;29(5):1161. 10.3390/molecules29051161 PMC10935070 38474673

[8] Gajera D, Trivedi V, Thaker P, Rathod M, Dharamsi A. Detailed review on gestational diabetes mellitus with emphasis on pathophysiology, epidemiology, related risk factors, and its subsequent conversion to type 2 diabetes mellitus. Horm Metab Res. 2023;55(5):295–303. 10.1055/a-2061-9441 36963428

[9] Lu W, Hu C. Molecular biomarkers for gestational diabetes mellitus and postpartum diabetes. Chin Med J (Engl). 2022;135(16):1940–51. 10.1097/CM9.0000000000002160 PMC9746787 36148588

[10] Entezari M, Hashemi D, Taheriazam A, Zabolian A, Mohammadi S, Fakhri F, et al. AMPK signaling in diabetes mellitus, insulin resistance and diabetic complications: A pre-clinical and clinical investigation. Biomed Pharmacother. 2022;146:112563. 10.1016/j.biopha.2021.112563 35062059

[11] Aghaei-Zarch SM. Crosstalk between MiRNAs/lncRNAs and PI3K/AKT signaling pathway in diabetes mellitus: Mechanistic and therapeutic perspectives. Noncoding RNA Res. 2024;9(2):486–507. 10.1016/j.ncrna.2024.01.005 PMC10950585 38511053

[12] Guven B, Akdemir Y. The change pattern in serum G protein-coupled estrogen receptor-1 (GPER1) levels during pregnancy with and without gestational diabetes mellitus. Horm Mol Biol Clin Investig. 2021;43(2):207–10. 10.1515/hmbci-2021-0023 34787384

[13] Mlyczyńska E, Myszka M, Kurowska P, Dawid M, Milewicz T, Bałajewicz-Nowak M, et al. Anti-apoptotic effect of apelin in human placenta: Studies on BeWo cells and villous explants from third-trimester human pregnancy. Int J Mol Sci. 2021;22(5):2760. 10.3390/ijms22052760 PMC7967155 33803239

[14] Peng C, Feng Z, Ou L, Zou Y, Sang S, Liu H, et al. Syzygium aromaticum enhances innate immunity by triggering macrophage M1 polarization and alleviates Helicobacter pylori-induced inflammation. J Funct Foods. 2023;107:105626.

[15] Chen X, Huang J, Peng Y, Han Y, Wang X, Tu C. The role of circRNA polyribonucleotide nucleoside transferase 1 on gestational diabetes mellitus. Cell Mol Biol. 2022;68(6):148–54. 10.14715/cmb/2022.68.6.24 36227662

[16] Xie J, Li L, Xing H. Metabolomics in gestational diabetes mellitus: A review. Clin Chim Acta. 2023;539:134–43. 10.1016/j.cca.2022.12.005 36529269

[17] Bukhari I, Iqbal F, Thorne RF. Editorial: Relationship between gestational and neonatal diabetes mellitus. Front Endocrinol (Lausanne). 2022;13:1060147. 10.3389/fendo.2022.1060147 PMC9616566 36313786

[18] Ye W, Luo C, Huang J, Li C, Liu Z, Liu F. Gestational diabetes mellitus and adverse pregnancy outcomes: Systematic review and meta-analysis. BMJ. 2022;377:e067946 (1–13). 10.1136/bmj-2021-067946 PMC9131781 35613728

[19] Norris GA, Tsai AC, Schneider KW, Wu YH, Caulfield T, Green AL. A novel, germline, deactivating CBL variant p.L493F alters domain orientation and is associated with multiple childhood cancers. Cancer Genet. 2021;254–255:18–24. 10.1016/j.cancergen.2021.01.008 33550024

[20] Zheng Y, Zhu N, Wang J, Zhao N, Yuan C. Crocetin suppresses gestational diabetes in streptozotocin-induced diabetes mellitus rats via suppression of inflammatory reaction. J Food Biochem. 2021;45(9):e13857 (1–11). 10.1111/jfbc.13857 34309046

[21] Kadam I, Dalloul M, Hausser J, Huntley M, Hoepner L, Fordjour L, et al. Associations between nutrients in one-carbon metabolism and fetal DNA methylation in pregnancies with or without gestational diabetes mellitus. Clin Epigenetics. 2023;15(1):137. 10.1186/s13148-023-01554-1 PMC10464204 37633918

[22] Zheng XD, Huang Y, Li H. Regulatory role of Apelin-13-mediated PI3K/AKT signaling pathway in the glucose and lipid metabolism of mouse with gestational diabetes mellitus. Immunobiology. 2021;226(5):152135. 10.1016/j.imbio.2021.152135 34521048

[23] Hung TH, Wu CP, Chen SF. Differential changes in Akt and AMPK phosphorylation regulating mTOR activity in the placentas of pregnancies complicated by fetal growth restriction and gestational diabetes mellitus with large-for-gestational age infants. Front Med (Lausanne). 2021;8:788969. 10.3389/fmed.2021.788969 PMC8685227 34938752

[24] Liu H, Tang T. MAPK signaling pathway-based glioma subtypes, machine-learning risk model, and key hub proteins identification. Sci Rep. 2023;13(1):19055. 10.1038/s41598-023-45774-0 PMC10625624 37925483

[25] Lees JA, Dias JM, Rajamohan F, Fortin JP, O’Connor R, Kong JX, et al. An inverse agonist of orphan receptor GPR61 acts by a G protein-competitive allosteric mechanism. Nat Commun. 2023;14(1):5938. 10.1038/s41467-023-41646-3 PMC10517971 37741852

[26] Liu H, Li Y. Potential roles of cornichon family AMPA receptor auxiliary protein 4 (CNIH4) in head and neck squamous cell carcinoma. Cancer Biomark. 2022;35(4):439–50. 10.3233/CBM-220143 PMC12364253 36404537

